# Barriers and Facilitators to School-Based Parent Involvement for Parents of Urban Public Middle School Students

**DOI:** 10.1177/2158244014558030

**Published:** 2014-11-18

**Authors:** Kantahyanee W. Murray, Nadine Finigan-Carr, Vanya Jones, Nikeea Copeland-Linder, Denise L. Haynie, Tina L. Cheng

**Affiliations:** 1 Annie E. Casey Foundation, Baltimore, USA; 2 University of Maryland Baltimore, School of Social Work, Baltimore, MD, USA; 3 Johns Hopkins Bloomberg School of Public Health, Baltimore, MD, USA; 4 Trinity Washington University, Washington, DC, USA; 5 *Eunice Kennedy Shriver* National Institute of Child Health and Human Development, Baltimore, MD, USA; 6 Johns Hopkins University School of Medicine, Baltimore, MD, USA

**Keywords:** parent participation, middle schools, urban, violence

## Abstract

Using semistructured interviews, we explored barriers and facilitators to school-based parent involvement (SBPI) in a sample of predominately African American parents (*N* = 44) whose children attended urban public middle schools. Barriers to SBPI (e.g., perceptions of hostile parent–teacher interactions and aggressive, disrespectful students in the school) were more commonly reported than facilitators (e.g., child invitations for involvement). Findings suggest that parents’ motivations for engaging in SBPI may be undermined by a variety of barriers, resulting in low participation. Implications and tailored strategies for enhancing SBPI in this population are presented.

An extensive body of research has shown that parent involvement during middle school is associated with a range of positive academic outcomes including higher class grades and test scores and other school achievement outcomes (e.g., Grolnick & Slowiaczek, 1994; Gutman & Eccles, 1999; Hill et al., 2004; Hill & Tyson, 2009; Simons-Morton & Crump, 2003). In light of the benefits of parent involvement, policies to close the achievement gap have required school systems to develop comprehensive parent involvement and family– school partnership strategies (e.g., “No Child Left Behind Act,” 2002). Educators face unique challenges in their efforts to increase parent involvement as children transition from elementary to middle school. Compared with elementary schools, middle schools are both larger with respect to their physical size and their student body, and parents must interact with an increased number of teachers to stay abreast of their adolescents’ academic progress. These more impersonal school environments may present difficulties to parents as they attempt to develop new relationships and to understand how to be involved (Hill & Tyson, 2009; Seidman, Lambert, Allen, & Aber, 2003). Furthermore, young adolescents may discourage particular parent involvement activities when they perceive the activity as diminishing their autonomy (Stevenson & Baker, 1987). In addition, this is the age at which there are changes in both adolescents’ and parents’ beliefs about the boundaries of parental authority, which then leads adolescents to engage their parents less and parents to decrease their engagement with their children (Daddis, 2011).

The impact of school and developmental transitions on parent involvement is compounded by a multitude of school and parent factors that serve as barriers to middle school parent involvement (e.g., Eccles & Harold, 1993; Gonzalez-DeHass & Willems, 2003; Kim, 2009). African American parents with low incomes and low educational attainment face considerable parent involvement barriers (Halle, Kurtz-Costes, & Mahoney, 1997; Koonce & Harper, 2005; Trotman, 2001; Williams & Sanchez, 2013), and may experience these barriers to a greater extent than more advantaged parents or White parents do (Frew, Zhou, Duran, Kwok, & Benz, 2012; Griffith, 1998). In the current study, we identify barriers to middle school parent involvement among a sample of predominately African American parents with low incomes and low levels of educational attainment whose children attend urban public middle schools. We also identify facilitators to parent involvement in middle school, factors that have been examined in only a handful of studies on African American parent involvement (e.g., Archer-Banks & Behar-Horenstein, 2008).

## What Is Parent Involvement?

Parent involvement represents parents’ commitment of resources and time to the academic sphere of their children’s lives. Epstein (1995) identified six forms of parent involvement: (a) establishing home environments that support learning, (b) facilitating effective communication between school and home, (c) helping the school and supporting students, (d) learning at home, (e) participating in school decision-making processes, and (f) working with other stakeholders (i.e., students, school staff, community) to strengthen the school. Scholars usually group these parent involvement activities into two broad categories: *home-based* parent involvement and *school-based* parent involvement (SBPI; Deplanty, Coulter-Kern, & Duchane, 2007; Deslandes & Bertrand, 2005; Pomerantz, Moorman, & Litwack, 2007). Home-based parent involvement includes practices related to children’s education that take place outside of the school, usually within the home. These practices may be directly related to schoolwork, including assisting with homework, responding to children’s academic choices, and talking about academic issues (Eccles & Harold, 1993). SBPI occurs when parents actually make contact with the school and includes participating in general school meetings, communicating with teachers and administrators, attending school events, and volunteering at the school (Herrold & O’Donnell, 2008).

Researchers have also proposed that parents’ positive attitudes about education and their communication of expectations concerning academic achievement to their children represent additional components of parent involvement (Grolnick & Slowiaczek, 1994; Hill & Tyson, 2009). Hill and Tyson (2009) identified *academic socialization* as a form of parent involvement examined in the literature. Academic socialization includes parenting practices such as communication of expectations about educational attainment, cultivating academic and career aspirations, connecting schoolwork and current events, and discussing learning techniques with children (Hill & Tyson, 2009). In their meta-analysis on the extant research on middle school parent involvement, Hill and Tyson (2009) found that, although academic socialization was the strongest predictor of academic success, SBPI has also been associated with academic achievement and other measures of doing well in school. However, the benefits of these forms of parent involvement may not be widely realized in the middle school years. As mentioned previously, parent involvement declines in middle school (Herrold & O’Donnell, 2008) compared with elementary school, perhaps due to diminished opportunities (Dauber & Epstein, 1993; Gonzalez-DeHass & Willems, 2003). The identification of barriers and facilitators to parent involvement presents an opportunity to inform the development of strategies to increase middle school parental engagement, particularly among populations at greatest risk for low involvement.

## Identifying Barriers and Facilitators to Parent Involvement

Central to the identification of barriers and facilitators is a focus on factors that influence parents’ decisions to engage in parent involvement. The Hoover-Dempsey and Sandler model of the parent involvement process (Hoover-Dempsey, Bassler, & Brissie, 1997; Hoover-Dempsey & Sandler, 1995; Walker, Wilkins, Dallaire, Sandler, & Hoover-Dempsey, 2005) describes the specific processes that influence parents’ decisions to engage in parent involvement. This model of parent involvement process also explicates how they contribute to the forms of parent involvement implemented and to child outcomes. The current study focuses on the first two levels of this model, which describe the processes that influence parents’ decisions to engage in parent involvement and how parents become involved (i.e., the forms of parent involvement). Hoover-Dempsey and colleagues posit that parents’ decisions to engage in parent involvement are influenced by three motivational factors: (a) motivational beliefs, (b) parents’ perceptions of invitations to become involved, and (c) parents’ personal life context. We organize the review of the literature on barriers and facilitators to parent involvement by these three motivational factors.

Evidence suggests that African American parents, especially those of lower socioeconomic status (SES), may experience greater barriers to parent involvement than more advantaged parents or White parents do (e.g., Griffith, 1998). This highlights the salience of race as a potential factor shaping parent involvement. Thus, the literature review additionally identifies studies regarding how parents’ perceptions of racism may influence motivational factors for parent involvement. This inclusion of studies related to parents’ experiences with and perceptions of racism in the schools is in line with Critical Race Theory (CRT; Delgado & Stefancic, 2001), which has as a central tenet the notion that racism is endemic to American society. CRT has been used to analyze aspects of education such as instruction and curriculum through a lens that recognizes the pervasiveness of racism in schools and seeks to understand how racism shapes school policies and practices (Ladson-Billings, 1998).

## Motivational Beliefs

Motivational beliefs are first determined by parental role construction or parents’ attitudes and beliefs about their role as a parent in fostering their child’s educational success (Hoover-Dempsey et al., 1997; Walker et al., 2005). Parental role construction represents parents’ beliefs about what they *should do* regarding parent involvement (Hoover-Dempsey et al., 2005, Walker et al., 2005), and there is evidence that parental role construction is an important motivational factor for parents of diverse ethnic and cultural backgrounds (see Hoover-Dempsey et al., 2005, for a review). Other motivational factors are thought to translate into the parent’s taking action to become involved. This is particularly true of the second motivational belief: parents’ self-efficacy. This refers to parents’ belief that they are capable of helping their child achieve in school (Hoover-Dempsey et al., 2005, Walker et al., 2005). Parents’ self-efficacy for involvement may be a barrier to parents of low SES. For example, parents of limited educational backgrounds may lack the confidence to interact with teachers and navigate the school (Kim, 2009; Koonce & Harper, 2005). For low-income African American parents, perceptions of racism as well as their own negative school experiences may shape their self-efficacy and serve to distance them from schools (Van Velsor & Orozco, 2007).

## Perceptions of Invitations to Become Involved

Parents’ perceptions of invitations to become involved include specific invitations from the child. Child invitations for involvement may be both explicit (e.g., child asking parent to help with a fund-raiser) or implicit (e.g., parent observes that her child is struggling with a class and talks with the teacher; Walker et al., 2005). Invitations may also originate from the school through specific teacher invitations (e.g., teacher invites the parent to volunteer in a classroom) and general invitations for involvement from the school (Walker et al., 2005). There is evidence that some teachers may not invite parent involvement because of their frustration with low-achieving, low SES students (Eccles & Harold, 1993; Van Velsor & Orozco, 2007) or because they view the family as the source of their students’ achievement problems (Griffith, 1998; Trotman, 2001). Common misunderstandings include teachers’ negative perceptions about the efficacy and capacity of low-income parents and teachers’ beliefs in the effectiveness of parental involvement with this population (Kim, 2009). Parents are more likely to participate in school activities when they feel empowered by their interactions with the school staff (Baker, 1997). However, power differentials related to educational achievement and professional expertise may lead to unequal relationships between parents and school staff, thereby marginalizing low-income parents instead of empowering them (Barton, Drake, Perez, St. Louis, & George, 2004; Khan, 1996). Sometimes African American parents’ lack of confidence in their skills and capacity to interact with teachers (Lareau & Shumar, 1996) and perceptions of racism may further distance them from the schools even when invitations to the school are given (Koonce & Harper, 2005).

General invitations for involvement from the school relate to the general atmosphere or climate of the school (Walker et al., 2005). Establishing a welcoming school climate and effectively publicizing school events to parents are examples of ways schools invite parental involvement (Green, Walker, Hoover-Dempsey, & Sandler, 2007; Hoover-Dempsey & Sandler, 1997). For African American parents, a positive school climate is an important component of general school invitations for involvement. In a qualitative study of African American parents, Archer-Banks and Behar-Horenstein (2008) found that, although parents viewed parent involvement as important, the school environment (particularly school personnel’s expectations, practices, and policies) influenced their level of involvement. In addition, research suggests a responsive middle school environment could eliminate barriers existing between middle schools and African American parents (e.g., teacher’s low expectations for children’s academic potential and parental involvement; Archer-Banks and Behar-Horenstein, 2008; Overstreet, Devine, Bevans, & Efreom, 2005).

School violence is one dimension of school climate that has particular relevance to parents whose children attend public schools in urban communities. Urban schools have been shown to have higher levels of violent incidents in schools compared with suburban and rural schools (Neiman, 2011; Weishew & Peng, 1993). Although the links between school violence and parent involvement have not been widely examined, existing evidence suggests that school safety is associated with greater levels of parent involvement (Griffith, 1998; Kandakai, Price, Telljohann, & Wilson, 1999). School violence as a factor influencing parent involvement has broad implications because school violence is a national problem (Schonfeld, 2006). According to the most recent published findings of the national School Survey on Crime and Safety, approximately 73% of schools reported at least one violent incident at the school during the 2009–2010 school year (Neiman, 2011). Moreover, both the percentage of schools reporting student bullying daily or at least once a week and the rate of violent incidents was higher for middle schools than for elementary and high schools (Neiman, 2011). Examining the extent to which school violence, conceptualized as a specific component of the school climate, hinders or fosters parent involvement will add to the knowledge base regarding the role of school invitations in motivating parent involvement.

## Personal Life Context

Personal life context refers to parents’ skills and knowledge and the perceived time and energy parents can expend to become involved (Walker et al., 2005). Research suggests that the personal life context of low SES parents may present a plethora of barriers to parent involvement. For example, parents with low educational attainment may lack the requisite sets of skills and knowledge to assist their children with assignments especially beyond the elementary school grades (Trotman, 2001). Work often serves as a barrier for low-income parents to devote time to attend school meetings, volunteer at the school, or participate in other parent involvement activities (Mannan & Blackwell, 1992; Van Velsor & Orozco, 2007). Although work affects the ability of parents to participate in SBPI activities regardless of income group, work barriers differentially affect low-income parents. Low-income parents are more likely to have inflexible work schedules, multiple jobs, and/or positions without paid leave benefits (Mannan & Blackwell, 1992; Van Velsor & Orozco, 2007).

Limited resources, such as lack of transportation, have also been shown to hinder SBPI (Reglin, King, Losike-Sedimo, & Ketterer, 2003; Williams & Sanchez, 2013). Limited resources, moreover, hinder low-income parents’ ability to address the basic needs of children and other relatives with special needs (e.g., old parents) contributing to further time constraints that negatively affect low-income parents’ involvement (Baker, 1997). In addition to financial and time constraint barriers, low-income parents may also experience psychological barriers. For example, low-income parents who struggle to provide for their families’ basic needs may experience negative mental health effects including depression, which may limit parents’ capacity to engage in school activities (Van Velsor & Orozco, 2007).

## The Present Study

The purpose of this study was to explore barriers and facilitators to SBPI in a sample of predominately African American parents living in low-income urban communities whose children attend public middle schools. One of the goals of this qualitative study was to understand parental school engagement in an effort to inform parent–school collaboration efforts. We used the Hoover-Dempsey and Sandler model of parent involvement processes as an organizing framework to understand how barriers inhibit and facilitators foster parents’ motivation for involvement. In previous studies, researchers have largely relied on teachers and administrators to identify barriers and solutions to improving educational outcomes through enhanced parent involvement (Barton et al., 2004; Koonce & Harper, 2005) and only a handful of studies have examined the viewpoint of parents (e.g., Williams & Sanchez, 2013). This study contributes to the literature by examining the perspectives of parents and other caregivers who are predominately African American and reside in urban, low-income communities.

## Method

### Participants

Between October 2005 and July 2006, we conducted semistructured interviews with parents who previously consented to participate in the Steppin’ Up study (2004–2007), an investigation testing the impact of a violence prevention curriculum on early adolescent aggressive behaviors at three public middle schools in Baltimore City. In the academic school year prior to this study, two of the three participating schools were on probation for the federal designation of “persistently dangerous,” a label based on the numbers of student expulsions and suspensions for violent offenses (Maryland State Department of Education, 2007b). These two schools are also located in communities characterized by poor indicators of child health and safety including substantial numbers of juvenile arrests (Baltimore Neighborhood Indicators Alliance, 2014). All three schools served significant numbers of low-income students; between 75% and 89% of students qualified for free or reduced-cost school lunch during the 2005–2006 school year (Maryland State Department of Education, 2006). Although data regarding the race/ethnicity of personnel at each school are unavailable, system-level data indicate that 59.7% of Baltimore City Public School teachers were African American, 35.6% were White, and 4.7% were categorized as other during the 2005–2006 school year (Maryland State Department of Education, 2007a).

The original child eligibility criteria included (a) first-time sixth grader and (b) not in self-contained special education classes. The children of participants in the qualitative study were either in the seventh grade (enrolled in the prior year) or sixth grade (enrolled in the current year) and were still participating in the Steppin’ Up study’s follow-up assessments. Of the 857 parents eligible, 513 parents (60% participation rate) consented to participate in the larger Steppin’ Up study at the time of the qualitative study. The Institutional Review Boards of the Johns Hopkins University and the National Institute of Child Health and Human Development (NICHD) and the city school district review board approved this study.

Recruitment for the qualitative study was targeted to parents of seventh graders at one school (first cohort) and parents of sixth graders at all three schools (second cohort; *N* = 381). Using a randomized contact list, staff contacted parents through a combination of telephone calls and letters. Blocks of 10 parents at a time were contacted until the recruitment goal was met. When staff documented 10 consecutive unanswered phone calls, a visit was made to the parent’s home. If no one answered the door, a postcard was left with instructions on how to contact staff. Parents had to be English speaking to participate. Fifty-one parents were reached and asked to participate. A total of 44 parents agreed to participate (30 mothers, 5 fathers, and 9 other caregivers, including grandmothers and aunts). Although the contact list was randomized, the 44 participants represent individuals who self-selected to participate. A little more than one half were parents of sixth graders (*n* = 23), and the remaining were parents of seventh graders (*n* = 21). On average, parents were 41 years of age. The sample was predominately African American (*n* = 39), followed by White (*n* = 2), Latino (*n* = 1), Pacific Islander/Native Hawaiian (*n* = 1), and Other (*n* = 1). Most participants (63%) had a high school diploma/GED or less, and 50% reported incomes of less than US$15,000 per year.

### Data Collection

Three interviewers were researchers with prior experience conducting semistructured interviews, and the fourth interviewer was a postbaccalaureate research intern with some prior research experience. Prior to fielding, the two most experienced interviewers led a 2-hr training on the protocol and interview techniques. Researchers alternated roles as interviewer and notetaker, with the two most experienced researchers conducting the initial interviews to facilitate training. Continued supervision and weekly meetings between interviewers and senior staff ensured interview protocol adherence.

Researchers developed a semistructured interview guide to examine aims associated with the larger adolescent aggression study and, to a lesser extent, secondary aims associated with the current parent involvement study. Using this interview guide, researchers asked parents to discuss their views about violence, what they communicate to their children about violence and fighting, and what parents, schools, and communities can do to keep their children safe. Parents were also asked a series of questions about their current involvement in their child’s education and school. The interview guides were pilot tested with three parents recruited to the Steppin’ Up study. All interviews were digitally recorded and the notetaker wrote down participant responses, capturing impressions from body language as well as providing a backup data collection measure. The interviewer and notetaker met following the interview to debrief and complete a field interview observation form. Although the majority of interviews were held in parents’ homes, a small number of parents requested their interview take place at a community location (i.e., a private room at the child’s school). The interviews lasted about 1 hr and parents were provided financial remuneration for their time.

### Data Analysis

We compared the transcribed and recorded interviews for accuracy and completeness. Parent interview transcript files were uploaded into hyperRESEARCH 2.7 (HyperRESEARCH, 2009). We used a grounded theory approach for data coding (Strauss & Corbin, 1998). These data were coded by looking for themes that emerged from participants’ statements. A team of three researchers identified themes using the first 12 transcribed parent interviews. First, an experienced primary coder (D.H.) with expertise in qualitative research methodology open coded and consulted with two experienced qualitative coders Vanya Jones (V.J.) and Nikeea Copeland-Linder (N.C.). A coding manual was developed based on the initial 12 interviews and modified as subsequent interviews were coded. Each new idea generated a code; similar codes were grouped by themes. The experienced primary coder Denise L. Haynie (D.H.) trained three postbaccalaureate research interns and a doctoral student [Kimberly Chambers (K.C.), Amanda McEnery (A.M.), Elizabeth Noelcke (E.N.), Kantahyanee Murray (K.M.)], and all five individuals coded the transcripts. We utilized a double coding approach for each transcript to improve reliability. Two coders were responsible for coding a transcript and then meeting to compare every instance of coded text. The two coders achieved consensus on all instances of coding discrepancies by discussing the merits of the codes selected. When coders could not agree on the appropriate code for a particular text, these instances of text were noted and discussed in coding progress meetings led by the experienced primary coder (D.H.) who resolved all coding disagreements. Codes relevant to parent involvement in the child’s education were examined in the current study. The overarching themes represented by these codes are shown in Table 1. The Hoover-Dempsey and Sandler model of parent involvement processes was applied to the themes as an organizing framework.

## Results

Themes related to the three motivational factors for parent involvement proposed in the Hoover-Dempsey and Sandler model of parent involvement processes are described in the following sections. The three motivational factors are (a) parents’ motivational beliefs, (b) parents’ perceptions of invitations for involvement from others, and (c) parents’ perceived life context. First, we provide a description of the themes related to parents’ motivational beliefs. Next, we organize the remaining themes that describe parents’ perceptions of invitations for involvement from others and parents’ perceived life context under two sections: facilitators and barriers. Some themes included both facilitators and barriers. We organized these themes in the barriers section because the findings were predominately those that described impediments to engagement in SBPI.

### Parents’ Motivational Beliefs

#### Parental role construction

Nearly all parents indicated that being involved in their child’s school was an important role that parents should play in their child’s education. The following parent’s statement exemplifies this attitude:

[Parents] certainly play the most important role [in their children’s education], I think. And, we should always be active in everything that’s going on with them more than just checking homework and just making sure that the homework is done. (Mother, age 48)

Volunteering (e.g., aiding teachers in the classroom and chaperoning field trips) and attending PTA and similar school meetings were frequently mentioned as SBPI activities that parents should be a part of at their child’s school. However, the majority of parents reported low levels of actual engagement in volunteering and attending school meetings. One mother explained, “Every so often I’ve been to a PTA meeting or two. And I’ve spoken to my child’s teacher once or twice. But not as often as I think it should be, as often as I would like to be” (Mother, age 48).

Nearly three quarters of parents discussed the importance of supervising their child’s academic progress and social behavior. For some parents, this belief translated into SBPI practices. For example, one third of parents reported going to the school to check up on their child. This involved observing their child in the classroom or other locations in the school (e.g., lunchroom or cafeteria).

I’ll go up to the school to check his progress. I will peek in on him, even if I don’t let him see me. I will always talk to the teachers, but I just peek my head in to make sure he’s sitting down at his desk. (Mother, age 30)

In addition to motivational beliefs, parental involvement practices are also influenced by child invitations for involvement, teacher invitations for involvement, general school invitations for involvement, and parents’ perceived life context. In the next two sections, we describe the facilitators and barriers related to these components of the parent involvement process model.

### Facilitators

#### Child invitations for involvement

Child invitations for involvement are one way that parents perceive they are invited to become involved. In this sample, explicit and implicit child invitations for involvement contributed to parent engagement in SBPI. For example, three parents mentioned visiting the school to follow-up on their child’s claim that a teacher was treating them inappropriately (e.g., unfair punishment or harsh reprimands).

If he saying he’s having trouble with a teacher, I want to see for myself, how does that teacher come across to me when I’m talking to him or her. So that would be one reason I would go up [to the school]. (Grandmother, age 61)

Five parents indicated implicit child invitations for involvement that involved initiating communication with teachers to address problems related to their child’s academic progress or behavioral problems. One grandmother described talking with teachers about her child’s conflict with another student at the school:

I went down to [the school], but they weren’t for pleasant reasons. I went down there because I tried to avoid—you know, I could see an incident blowing up, blowing up with [my daughter] and the girls down there. I went down there to talk to the teacher about it. (Grandmother, age 66)

Teacher-specific invitations for involvement and general school invitations for involvement are the other ways parents perceive they are invited to become involved. In this sample, the themes that emerged for these two components of the parent involvement process model represented barriers to parent involvement.

### Barriers

#### Teacher-specific invitations for involvement

Teacher invitations for involvement were infrequently mentioned. When teacher invitations for involvement were extended, they were generally to address the child’s disruptive behavior problems. One parent described her perception that teacher invitations for involvement were both rare and behavior-problem focused:

You’ve got to talk to these teachers. Just be involved. Because a lot of the times, teachers don’t even—if your child does not have a behavior problem, but they could still be in the class not doing what they’re supposed to do, you won’t get a phone call. The only time they give you phone calls is if it’s a behavior problem that’s affecting them. (Mother, age 26)

This quote illustrates some parents’ perceptions that teachers do not invite parents to become involved when their child has difficulties not related to problem behavior in the classroom. This absence of teacher invitations to become involved impedes SBPI; that is, teachers’ failure to make parents aware of particular problems with their children limits parents’ opportunities for SBPI.

Ten parents reported teacher invitations to come to the school because their child was involved in a conflict (typically a fight) with another child or exhibited behavior problems in class. In these cases, parents were visiting the school to participate in parent–teacher conferences that were often mandatory.

Approximately one half of parents indicated having negative impressions of teachers in the school and generally discussed unfriendly and hostile interactions with teachers. Some of these parents reported instances when teachers were disrespectful to or inappropriately communicated with their children.

#### General school invitations for involvement

Most parents indicated that their children’s school offered opportunities for SBPI. However, parents indicated that opportunities for involvement were not communicated in a timely, organized fashion. For example, some parents reported not learning about opportunities including school assemblies and meetings until it was too late for them to rearrange their schedule or until after they had occurred. One mother said,

Communication is lackadaisical and next to none. When she’s in trouble, they’re quick to pick up the phone, but I don’t hear nothing about PTA meetings … [T]hey’re going on a field trip and they sent the permission slip home like two, maybe three days before the trip. I’ve seen a few pieces of paper from time to time, but again, anything regular or anything that I can like put my hands on and make a calendar from, it doesn’t happen. (Mother, age 33)

Although two parents indicated that their child failed to give them flyers and other materials publicizing activities, the majority of parents attributed poor communication to inadequate organization and communication channels at their child’s school. Other less commonly discussed SBPI issues included parents’ perception that the school offered no SBPI opportunities, offered them at inconvenient times, such as during the workday, and an impression that the PTA was ineffective.

Nearly all parents mentioned having a negative impression of one or more aspects of the school climate at their child’s school. More than one third of parents mentioned having an overall negative impression of the school, including their perceptions of the school’s discipline and safety problems and criticisms of the administration’s inability to effectively address school challenges. Furthermore, 16 parents expressed negative impressions of students in their child’s school. These parents generally discussed the high levels of aggressive and disrespectful student behavior in the schools. One father stated,

The teachers and administration had no control at all. It was almost like a three-ring circus, kids cussing, threatening, fighting, running, throwing stuff; you name it, knocking people out of the way. No respect. (Father, age 53)

Parents’ displeasure with aggressive and disrespectful students was reported as an obstacle to engaging in SBPI. One parent said, “I don’t want to go [to the school] because sometimes you can’t deal with other people’s children … They [are] disrespectful and they get smart with you in a minute” (Mother, age 32). Furthermore, other parents in the child’s school were described in a negative light. One mother described how she perceived other parents as antagonistic and difficult to engage when resolving child behavior-related problems:

When you do have little problems with children at the school and you try to go to this parent, you don’t want to go—they get mad when you approach their child when [they are] not around. But then [these parents are] nowhere to be found when I need to talk … about my child’s problem. (Mother, age 26)

Ten parents reported wanting little or no contact with other parents, an attitude that impedes engagement in SBPI. Parents’ desire to avoid potentially negative interactions was the most common reason parent-to-parent contact was not wanted. Although parents generally reported not knowing other parents, one half were interested in meeting other parents. Parents’ reasons for wanting to get to know the parents of their children’s friends included having a way to keep track of their child’s whereabouts and activities when unsupervised and building relationships to facilitate communication about issues relevant to their child’s schooling.

### Parents’ Perceived Life Context

#### Time and energy

Work and scheduling issues were the most frequently reported barriers to SBPI. One common response by parents was that they had no time to participate in SBPI consistently or at all because of their demanding work schedules and the absence of paid leave benefits. Therefore, they were unable to or could not afford to take time off to participate in school activities. Often the combination of work issues and family responsibilities synergistically created more obstacles to SBPI. One parent explained,

I mean, basically [parents] just don’t have time, unless they go [to the school] like early in the morning or whatever … I really don’t have time because when I get off work or go to my other job, or have to come home, clean up. Like right now, I’m washing, and I have to cook dinner. I really don’t have time. But I would like to, I really would, but I’m not rich. (Mother, age 45)

Despite such multifaceted work and scheduling challenges, a small number of parents described strategies or sacrifices made to overcome these SBPI barriers. One mother said,

So yes, I do have to forgo some sleep sometimes. You know, when I was working during the day, there were days when I would let my boss know the day before. “Look, I’m going to be late coming in because I have to go and visit my child’s school.” (Mother, age not disclosed)

#### Skills and knowledge

There were no study findings directly related to parents’ skills and knowledge (or attitudes regarding their skills and knowledge) as it pertains to engagement in SBPI. Rather, the findings related to parents’ skills and knowledge were directly relevant to home-based parent involvement (e.g., attitudes that endorsed providing academic assistance, linking their children to trusted family members and trusted adults in the community who could provide academic help).

## Discussion

Guided by the Hoover-Dempsey and Sandler model of parent involvement processes (Hoover-Dempsey et al., 1997; Walker et al., 2005), we identified barriers and facilitators to SBPI among parents who lived in low-income urban communities and whose children attended three public middle schools. Our findings support previous research demonstrating links between motivational factors (i.e., motivational beliefs, invitations for involvement, and personal life context) and the frequency and types of parent involvement implemented (Deslandes & Bertrand, 2005; Green et al., 2007; Hoover-Dempsey et al., 2005). In the current study, motivational beliefs for parent involvement were expressed in the form of parents’ positive role construction regarding their function to support their child’s educational endeavors. However, the quality of invitations for involvement by teachers and the school as well as parents’ personal life context presented a number of barriers. One important contribution of this study is the finding that students’ aggressive behavior in the school not only contributed to some parents’ negative perceptions of the school climate but in fact it also hindered their engagement in SBPI. Overall, the barriers identified help to explain why parents in the current study indicated infrequent involvement in SBPI activities.

### Motivational Beliefs

Parents overwhelmingly reported positive attitudes and beliefs regarding their role in fostering their child’s educational success. Consistent with previous research examining parental role construction (Baker, 1997; Drummond & Stipek, 2004; Sheldon, 2002), the parents in the current study expressed that they have a role in their child’s education and were interested in being involved in their child’s education at home and at school. In the current study, parents’ perceptions of their self-efficacy, the second component of motivational beliefs, were not reported. Some research suggests that parents’ self-efficacy may be low among parents with limited educational attainment (Kim, 2009; Koonce & Harper, 2005). However, in a sample of parents of diverse ethnic and SES backgrounds, Green et al. (2007) found a negative association between self-efficacy and SBPI. This finding suggests that parents who are less confident about their capacity to help their child in school may interact with the school more often to obtain support (Green et al., 2007). Additional research in this area may help to elucidate the relationship between self-efficacy and SBPI specifically among populations more similar to the sample in the current study.

### Parents’ Invitations for Involvement

Parents reported invitations for SBPI from their children and their teachers. Invitations from children were facilitators to SBPI in that they involved parental initiation of contact with teachers and other school staff, often to address problems including their child’s difficulties with schoolwork or behavior. Most parents indicated that their initiation of contact with teachers was less common than teacher’s invitations for SBPI involvement. Yet, parents indicated that teacher’s invitations for SBPI involvement were often mandatory parent– teacher conferences. Regardless of the source of invitation for involvement, parents generally reported negative and sometimes hostile interactions with teachers and other school staff that can present barriers to future parent involvement that is up to their discretion. This finding is similar to previous research indicating that unfriendly and hostile relationships frequently characterize parent and school personnel interactions in predominately low-income, minority samples (Barton et al., 2004; Koonce & Harper, 2005).

Moreover, some parents in this sample noted instances when teachers were inappropriate or disrespectful to their children. Despite these negative experiences, parents expressed little reticence about interacting with teachers and staff. This finding contrasts that of previous studies that identify barriers such as parents’ lack of confidence when interacting with teachers and staff and perceived racism as hindrances to parent involvement (Kim, 2009; Koonce & Harper, 2005; Van Velsor & Orozco, 2007). Because perceived racism based on SES or race was not explicitly explored in the current study, the absence of barriers related to these factors is uncertain. The exploration of African American parents’ experiences with racism in their interactions with school personnel merits additional research. Qualitative research that allows African American parents to tell their stories about racism they have experienced in their children’s school reflects another central tenet of CRT (i.e., counterstorytelling; Delgado & Stefancic, 2001) and promises to yield rich data for critical analysis through a CRT lens.

Present study findings indicate that many parents held negative impressions of the school. Parents reported that general school invitations for SBPI about school events and meetings were poorly coordinated. Information about SBPI opportunities were communicated inconsistently or too late for parents to plan appropriately. This poor communication of SBPI opportunities may worsen parents’ perceptions of the school climate. Aggressive and disrespectful students dissuaded some parents from visiting the school, and interactions with other parents were viewed unfavorably. One finding of the study suggests that the school climates of the three middle schools in this study were not welcoming. Given that a welcoming school climate is an indicator of general school invitations for parent involvement (Green et al., 2007; Griffith, 1998; Hoover-Dempsey et al., 2005), an unfriendly school climate may have diminished parents’ perceptions of general invitations for parent involvement from the schools. Study findings suggest the centrality of student behavior in parents’ perceptions of the qualities of the school environment. In particular, the perceived school safety risks that stemmed from aggressive and disrespectful students seemed to repel some parents in the current study from visiting the school rather than encourage more frequent visits. This interpretation is in line with prior research indicating a positive association between school safety and parent involvement (Griffith, 1998).

It is also noteworthy that the current study described parents’ lack of contact with other parents at the school. This finding is consistent with research indicating that the social networks of low-income parents do not prominently include other parents at their children’s schools (Lareau & Shumar, 1996). This lack of social connection limits parents’ ability to learn information about the school or their children’s educational process and may play a role in parents’ decisions to engage in SBPI (Lareau & Shumar, 1996; Sheldon, 2002).

On the whole, the parent invitations for involvement findings demonstrate that the parents in this study generally had negative experiences with the schools. It is interesting to note that parents’ negative experiences with the school both fueled and thwarted SBPI. For example, the prospect of having hostile interactions with teachers reduced the appeal of SBPI for many parents; however, a child experiencing teacher disrespect and antagonism was a catalyst for SBPI. Similarly, the negative school climate repelled parents, yet at the same time, the deleterious school climate contributed to the ubiquitous nature of classroom conduct problems and peer conflicts. Thus, engaging in SBPI to address such problems was a common experience for a number of parents in the study. Such parent invitations for involvement findings exemplify the transactional nature of the interactions between parents, their children, teachers, administrators, as well as other students and their parents.

### Personal Life Context

Work and scheduling issues were the most commonly identified barriers. Parents indicated various scheduling issues that presented a challenge to school involvement including work, raising children, and family responsibilities (e.g., preparing dinner, picking up more than one child from school). These barriers are consistent with previous research on SBPI indicating that inflexible work schedules, multiple jobs, and the absence of leave benefits are tremendous obstacles to parental involvement for parents in single-headed households or of lower SES (Archer-Banks & Behar-Horenstein, 2008; Lareau & Shumar, 1996; Van Velsor & Orozco, 2007). Parents also conveyed how demanding work schedules and multifaceted family responsibilities diminish their physical energy, thus, inhibiting their ability to engage in SBPI. Some parents reported having the flexibility to rearrange work schedules for school events; however, they reported that their child’s school’s untimely communication of events prevented them from having enough time to rearrange their work schedules. Unlike previous research (Lareau & Shumar, 1996; Reglin et al., 2003), limited resources (e.g., lack of transportation) were mentioned infrequently as barriers to SBPI. The availability of resources such as transportation and child care was not probed in this qualitative study. Therefore, it is unclear why limited resources failed to emerge as a frequent barrier among parents in the current study.

Another element reported was that parents’ perceptions of their skills and knowledge in the context of SBPI were not reported. In a study of parents from diverse ethnic and SES backgrounds, skills, and knowledge were unrelated to SBPI (Green et al., 2007). However, other findings from the same study suggest that parents with low self-efficacy regarding their capacity to help their child in school may engage in SBPI more often to obtain support (Green et al., 2007). As mentioned above, parents’ self-efficacy for involvement may be a barrier to parents of low SES (Hoover-Dempsey et al., 2005, Walker et al., 2005). Parents’ confidence in engaging with the school may be influenced by their educational attainment (Kim, 2009; Koonce & Harper, 2005) and other indicators of knowledge and skills. Additional research on the role of self-efficacy in predicting SBPI should also incorporate measures of knowledge and skills as well as analyses that explore both direct and indirect relationships among these factors.

### Policy Implications

For schools, building strong parent–school partnerships requires practical steps that aim to enhance general school invitations and teacher invitations for involvement. Schools can also tailor SBPI activities to address the barriers experienced as a result of parents’ personal life context. For example, schools can help increase SBPI by implementing more reliable and timely methods of communication (e.g., utilization of social media or texting), scheduling school meetings and events at varied or multiple times, and soliciting parents’ ideas on other ways to overcome work- and scheduling-related barriers. In addition, school counselors, social workers, and other human service professionals can play a pivotal role in fostering positive parent–teacher relationships. On one hand, these professionals can educate teachers and other school staff about the positive role construction and other assets (i.e., problem-solving skills and access to resources in parents’ social networks) parents can bring to collaborative processes. On the other hand, these professionals can encourage parent visits by greeting and orienting them to SBPI opportunities, thus, boosting the positive aspects of the school climate. Given parents’ concern about aggressive students in the schools, offering opportunities to inform school safety promotion programs and policies may be a good approach to engaging parents in school improvement efforts. Finally, the parent concerns expressed about negative interactions with other parents and students highlights the need for activities that promote community building, shared goals, and positive interactions with the schools.

### Limitations

It is important to note that major school system policy changes since the study period have resulted in the implementation of new parent engagement initiatives at the local and state levels. Thus, compared with parents in the present study, current parents of students attending these middle schools may have a different impression of parent involvement barriers and facilitators. Study findings should be considered in light of several other limitations. First, only the perspectives of parents and other caregivers were examined. The perspectives of teachers and other school personnel in addition to parents may provide a more comprehensive understanding of issues and solutions. Second, the absence of specific interview questions regarding race as a potential barrier to parent involvement may mean that important race-based dynamics were not identified and explored. It is also unlikely that the attitudes and practices reported by the current study participants fully represent those of the parents who participated in the larger intervention study as well as the larger pool of parents whose children attended the three middle schools. Because our recruitment strategy primarily involved contacting parents by telephone, parents with disconnected or inconsistent telephone service were underrepresented in the study. This may have biased the sample toward including parents with relatively higher incomes and more resources. Although attempts were made to contact parents during both day and evening hours, parents who worked more than one job or with higher family management demands may have been more difficult to reach. Parents whom staff were unable to reach or who refused to participate may have been those experiencing more extensive barriers to SBPI. Findings should be interpreted with caution given the limited generalizability of the study findings.

### Conclusion

More barriers than facilitators emerged in this exploration of factors that inhibit or foster parents’ motivation for SBPI in a sample of predominately African American parents who have low incomes and whose children attend urban, public middle schools. The negative quality of parents’ interactions with teachers, the schools, other children and parents, as well as work and scheduling challenges were factors that hindered SBPI. In spite of this, the positive motivational beliefs parents expressed, parents’ responses to child invitations for involvement, and the interest of some parents to get to know other parents are foundations to build on for the development of stronger, parent–school partnerships characterized by collaboration and shared power (Powell & Batsche, 1997). Future research directions should include further examinations of the role of school climate (including factors related to students and other parents in the schools) in motivating parent involvement among African American parents of children attending middle schools with school safety risks. In predominately African American samples, an emphasis on understanding the role of racism in shaping policies that contribute to unsafe school climates may particularly help to inform initiatives to improve the school environment.

## Figures and Tables

**Table 1:** Overarching Themes Relevant to School-Based Parent Involvement Examined in the Current Study.

Themes	Refers to statements about parent’s …
Parent role in school	Beliefs and practices around supervising child’s education and homework, providing academic assistance, participating in PTA, volunteering at the school, and other activities to support children’s education.
Parent involvement in school	Practices and activities the parent does at the school, including going to the school to talk to teachers, making unannounced visits. Distinctions were made between teacher/staff-initiated contact and parent-initiated contact.
Parent contact with other parents	Interactions with other parents (i.e., limited to problem, little/none and would like more, little/none preferred).
Impression of school	Overall impressions of the school (i.e., positive to neutral, negative staff focused, negative student focused, negative parent focused, negative school facility focused, perception that school discipline is unfair/inconsistent).
Barriers/challenges to school involvement	Descriptions of factors that inhibit involvement in the child’s school, including schedule, work issues, transportation barriers. Student behavior and lack of opportunity at the school were also mentioned.
Challenges raising an early adolescent	Descriptions of the difficulties they face raising their sixth or seventh grader, including developmental issues, school issues, increased misbehavior or opportunities for misbehavior, and youth lack of disclosure about friends, school, peers, or problems.
